# The mediating effect of dietary patterns on the association between mother’s education level and the physical aggression of five-year-old children: a population-based cohort study

**DOI:** 10.1186/s12887-020-02126-5

**Published:** 2020-05-15

**Authors:** Wen-Chi Wu, Ching-I Lin, Yi-Fan Li, Ling-Yin Chang, Tung-liang Chiang

**Affiliations:** 1 Department of Health Promotion and Health Education, National Taiwan Normal University, 17 Xuzhou Road, Taipei, 100 Taiwan; 2 Department of Nutrition and Health Sciences, Kainan University, No. 1, Kainan Road, Luzhu District, Taoyuan City, 338 Taiwan; 3 Division of Clinical Chinese Medicine, National Research Institute of Chinese Medicine, Ministry of Health and Welfare, 11221 Room, No. 155-1, Section 2, Linong St, Beitou District, Taipei City, 112 Taiwan; 4 Institute of Health Behaviors and Community Sciences, School of Public Health, National Taiwan University, 17 Xuzhou Road, Taipei, 100 Taiwan; 5 Institute of Health Policy and Management, School of Public Health, National Taiwan University, 17 Xuzhou Road, Taipei, 100 Taiwan

**Keywords:** Dietary pattern, Physical aggression, Maternal education, Preschoolers

## Abstract

**Background:**

Relatively few studies have investigated the effects of diet on behavior problems among preschoolers, particularly, physical aggression. In addition, children raised by poorly educated mothers usually have a higher probability of developing negative outcomes. Additionally, highly educated mothers have a higher probability of providing more healthy foods for their children. Thus, mothers providing healthy foods might mitigate children’s behavior problems. The study aims to examine whether preschoolers’ dietary pattern, as a manipulable factor, mediates the association between maternal education level and physical aggression.

**Methods:**

Data came from the Taiwan Birth Cohort Study (TBCS), a nationally representative population-based cohort study, which included 18,513 five-year-old Taiwanese children. Mothers and primary caregivers reported the information on preschoolers’ physical aggression and food consumption at age 5 and maternal education level at age 6 months. Two dietary patterns, namely a healthy diet and a high-fat-sugar-salt (HFSS) diet, were retrieved by exploratory factor analysis. Mediation hypotheses were tested by a series of multiple regression models conducted using the PROCESS macro of SAS 9.4. All models were adjusted for children’s sex, parental marital status, household income, mental distress at age 5 and children’s physical aggression at age 3.

**Results:**

Maternal education positively linked to healthy dietary patterns (B = 0.014, *p* = 0.002) which was negatively associated with preschoolers’ physical aggression (B = -0.096, *p* = 0.013), and it is negatively related to the HFSS dietary pattern (B = -0.042, *p* = 0.002) which was directly positively associated with preschoolers’ physical aggression (B = 0.123, *p* = 0.008). The association between maternal education and preschoolers’ physical aggression was partially mediated by preschoolers’ healthy (B = -0.001, *p* < .001) and HFSS (B = -0.005, p = <.001) dietary patterns, respectively. The R-square of the mediation model is 0.178.

**Conclusions:**

Preschoolers’ dietary patterns directly associate with their physical aggression. In addition, mothers with poor education may provide less healthy foods and more unhealthy foods to their children, which may increase the level of physical aggression. The results imply partial mediating effects of dietary patterns between maternal education and physical aggression. It is suggested that a parent-based nutritional education program focusing on healthy meal preparation for poor educated mothers might be beneficial for preschoolers’ healthy development.

## Background

Physical aggression in early childhood is a common behavioral problem recognized by parents and teachers because it is easily observed as overt acts. The behavior not only affects the children’s performance but also their interactions with siblings or peers [1, 2]. Moreover, children with a higher level of physical aggression were more likely to develop adolescent physical aggression as well as anti-social and health risk behaviors [3, 4]. Thus, understanding the related factors of young children’s aggression is essential for physical aggression prevention and child health promotion.

Many factors impact physical aggression in early childhood [5]. One of the proximal factors raising considerable concern is diet [6, 7]. Diet has been associated with many conditions such as hyperactivity, attention deficiency, poor impulse control, conduct disorder, and aggressive behaviors [8–10]. Studies have found that some components of food may trigger behavioral problems, such as processed sugar, saturated fat, allergenic elements, and chemical pigments [7, 10]. However, the caveat of these studies is that they focused on the association of a single nutrient with behavior, while few studies have investigated the dietary patterns in relation to aggression, especially among preschoolers.

Instead of examining the single and relatively small effect of a nutrient on behavior, understanding the cumulative effects of multiple nutrients contained in a dietary pattern may be more effective for risk prevention and health promotion [11]. Forming a proper dietary pattern is vitally important for young children because they are in the developmental stages of life. Although relatively few studies have empirically examined the effect of dietary pattern on physical aggression in early childhood [9], dietary pattern has been associated with depression [12], autism [13], and attention deficit hyperactivity disorder [14]. Thus, to prevent and reduce preschoolers’ physical aggression, more evidence is necessary to understand the influences of different dietary patterns on physical aggression.

One important contextual factor of physical aggression in childhood is maternal education, that is, the highest level of schooling attended by mothers [15]. The theory of family functioning proposes that poorly educated mothers, who may usually possess less knowledge and fewer resources to raise children adequately, are less likely to supervise and discipline their children, which increases the likelihood that a child will develop aggression-related behaviors [15]. Maternal education may have a larger effect than paternal education due to mothers commonly being the primary caregivers for preschoolers, especially in disadvantaged families [16, 17]. Maternal education was found to be an influential factor in preschoolers’ physical aggression [18]. Furthermore, it is also a crucial predictor of high-level physical aggression from early childhood to adolescence [19].

Similarly, maternal education links to children’s dietary patterns because mothers are usually the primary food providers for the majority of children [20]. Research has indicated that children with a poorly educated mother had a higher score on the unhealthy dietary pattern [21]. Moreover, a highly educated mother may promote her children’s healthy diet through her own eating behavior [22].

From the results of the above studies, maternal education and diet are directly associated with physical aggression. In addition, maternal education can affect children’s problem behavior either independently or through diet. It is speculated that dietary pattern may be a potential mediator between maternal education and physical aggression. However, no study has investigated the dietary pattern as a pathway from maternal education to physical aggression. Due to the alterability of dietary patterns, it may be a feasible target for a behavior intervention strategy.

The present study aims to examine whether dietary patterns mediate the relationship between maternal education and physical aggression.

## Methods

### Participants

The study data came from the Taiwan Birth Cohort Study (TBCS), the first longitudinal study which follows up a nationally representative sample of children born in 2005 [23, 24]. The project team used birth certificate data from 2005 as a sampling frame, and conducted two-stage stratified random sampling methods for selecting participants. First, using the urbanization level and birth rates of 369 residential areas, we selected 89 areas by systematic random sampling methods. Second, there were 24,200 participants selected from the areas using the probability proportional to size method. The average sampling rate was 11.7%. TBCS aimed to document Taiwanese children’s health and developmental trajectories in order to investigate the influence of social environment on children’s health, and to examine how early events influence adult health by using the life course approach. Mothers or primary caregivers provided their information by face-to-face interviews when the subjects were 6 months, 8 months, 3 years and 5.5 years of age with response rates of 87.8, 94.9, 93.7, and 92.8%, respectively. The reasons for attrition included being too busy, low willingness to participate, moving house, etc.

The interviewers received a “letter to the participants” from the Director-General, Health Promotion Administration, Ministry of Health and Welfare, Taiwan. The letter elaborated on the purposes, sampling methods, confidential process and contact information of the administrator. Then, the participants obtained a letter of consent from the interviewers in person. After the interviewee fully understood their rights and obligations and signed the consent form, the interviewer started the interview.

This study included 19,721 subjects whose parents and primary caregivers completed the interview survey at 5.5 years old. We used their information which was measured at 6 months, 3 years old, and 5.5 years old. After excluding the participants with missing values, there were 18,513 participants (52.49% boys) analyzed in this study (retention rate = 93.9%). There were no differences between the analytic sample (*n* = 18,513) and the original sample (*n* = 19,721) in terms of sex, maternal education level, food consumption, mental distress, and physical aggression. However, those who remained in the analytic sample were significantly more likely to have parents who were married (90.67% vs. 87.63%) or parents with a higher education level (mean = 3.91 vs. 3.87; both *P* < 0·05).

### Instrument development

The TBCS instrument was developed according to the following process. First, the first version of the questions for measuring each concept, such as physical aggression, dietary pattern and mental distress, etc., was developed based on previous literature. Second, the first-version questionnaire was reviewed by experts to ensure its face validity and was revised according to the reviewers’ comments. Third, the revised-version questionnaire was used for the pilot study (*n* = 1620) for pre-testing all the questions, and the participants’ comments and feedback were further collected to revise the questionnaire into its final version. In addition, some concepts, such as aggression and mental distress, were excerpted for a small group two-week test-retest reliability construction (*n* = 18).

### Measurements

#### Maternal education level

Maternal education was measured based on the participants’ report of the highest level of education attained by the mother when the child was 6 months old, ranging from uneducated (coded as 0) to graduate school or above (coded as 17). One score unit represents 1 year of education the mother completed. For example, score 11 means the mother completed the second year of high school education but did not graduate from high school, and score 12 means the mother graduated from high school.

#### Food groups for dietary patterns

Children’s diet was measured by the reports of primary caregivers when their child was 5 years old. The respondents were asked “How many times does your child eat the following foods a week?” The options included never (score = 0), less than once per week (score = 1), once or twice per week (score = 2), three to five times per week (score = 3), and almost every day or every day (score = 4). The 11 food groups were presented: meats, seafood (such as fish and shrimp), beans/bean products, eggs, grains/starchy roots, vegetables, fruits, dairy products, burger/pizza/fried chicken, candy/cookies/cakes, and beverages/Coca-Cola/Soft drinks. The first eight food groups were developed based on the Daily Dietary Guidelines provided by Taiwan’s Ministry of Health and Welfare [25], and the other three groups were chosen because they are common foods that are high in fat, sugar or salt [26, 27]. These groups of foods were used for the Exploratory Factor Analysis (EFA) to extract dietary patterns based on the variable-centered approach.

#### Physical aggression

The measurement of physical aggression for five-year-old children was developed with reference to the scale developed by Tremblay et al. [1] and Cote et al. [19]. The respondents were asked, “Did your child exhibit any of these behaviors in the last month?” when their children were 5 years old. Three items were used to measure physical aggression, including beating others, fighting with others, and biting or kicking others. Scores on this 3-item scale ranged from 1 (never) to 5 (always). The level of physical aggression was measured by averaging the responses, with higher scores indicating a higher level of physical aggression (Cronbach’s α = 0.77). The correlation coefficient of two-week test-retest reliability is 0.82.

#### Covariates

Five covariates were included to account for the potential confounding in the association between parental education level, food consumption, and physical aggression. Children’s sex was coded as 1 = boy and 0 = girl. Parental marital status at age 5 was coded as 1 = married and 0 = not married. Children’s physical aggression at the age of 3 was measured with the same three items which were used to measure physical aggression at age 5 (Cronbach’s α = 0.77). The responses of the three items were averaged to indicate the level of children’s physical aggression at the age of 3. Children’s mental distress scale was developed with reference to internalizing items of the Behavioral Assessment Scale for Children [28] and the Brief Problem Monitor-Parent Form [29]. To save space in the questionnaire, three questions, namely “Your child looks sad or depressed for no special reason,” “Your child feels fearful or anxious because of small matters” and “Your child worries about things not being well-done,” were selected and revised by some experts specializing in child psychology, and the questions were rated on a 5-point scale (ranging from 1 to 5) at age 5 (Cronbach’s α = 0.61). The correlation coefficient of two-week test-retest reliability is 0.76. The mental distress level was constructed by averaging the responses to the three items, with higher scores indicating a higher level of mental distress. Household income was measured when the child was 6 months old by the respondents’ reports of the total family income in the last year, ranging from less than 100,000 NTD (coded as 1) to more than 3,000,000 NTD (coded as 8; 30 NTD ≒ 1 USD).

#### Analytical strategy

Dietary patterns were extracted by EFA. Descriptive statistics, including sample size, percentages, means, and standard deviations, were used to describe the characteristics of the study sample. Correlation analysis was conducted to determine the associations among maternal education, children’s dietary patterns, and children’s physical aggression, as well as covariates.

**Fig. 1:**
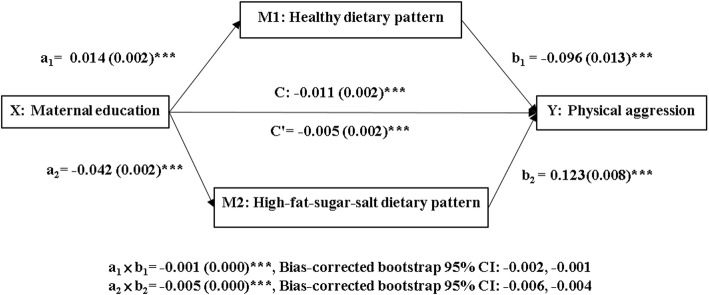
A mediation model of association between maternal education and children’s physical aggression through dietary patterns. a_1_ and a_2_ = effects of maternal education on a healthy dietary pattern and a high-sugar-fat-salt dietary pattern. b_1_ and b_2_ = effects of healthy and high-sugar-fat-salt dietary patterns on physical aggression. C = total effects of maternal education on physical aggression. C′ = direct effect of maternal education on physical aggression after adjustment for dietary patterns and covariates. a_i_ × b_i_ = mediation effects of dietary patterns on the relationship between maternal education and physical aggression. The parameter estimates were presented as B(SE). X: independent variable. Mi: mediators. ***: *p* < .001. CI: confidence interval

In order to examine whether children’s dietary patterns mediate the association between maternal education and preschoolers’ physical aggression, we conducted a parallel multiple mediator model proposed by Hayes [30]. A series of multiple regression models were used to examine the association between maternal education and preschoolers’ dietary patterns (Fig. 1, path a_1_ and a_2_), between preschoolers’ dietary patterns and their physical aggression (path b_1_ and b_2_), and between maternal education and preschoolers’ physical aggression (path c, total effect) after adjusting for covariates. The direct effect of maternal education on preschoolers’ physical aggression (path c’) after adjusting for preschoolers’ dietary patterns and covariates was also computed. The mediation effects, i.e., the product of the coefficients of paths a_i_ and b_i_ were determined by using a bootstrapping method with 10,000 resamples. The 95% bias-corrected bootstrap confidence interval (CI) was used to judge the significance of the mediation effect by not containing zero. All of the above analyses were conducted with the PROCESS macro version 2.2 of SAS 9.4. All models were adjusted for children’s sex, parental marital status, household income, mental distress at age 5 and children’s physical aggression at age 3.

## Results

### Dietary patterns

Exploratory Factor Analysis (EFA) using the original five-ordinal level of food groups with oblique rotation (Promax) was conducted to generate the latent structure of dietary patterns [31, 32]. The maximum likelihood method was used during iteration to estimate communalities. Diagonals on the correlation matrix are squared multiple correlations (SMC) [33]. The Kaiser–Meyer–Olkin value was 0.68. All analyses were conducted in the Statistical Analysis System software package version 9.4 (SAS Institute, Cary, NC, USA). Two factors were retained based on the retaining factors that accounted for greater than 75% of the common variance and had an eigenvalue greater than one. Eigenvalues of the weighted reduced correlation matrix are 1.82 and 1.23. The proportion of variance explained by each factor is 60 and 40%. Cumulative variance for the two factors is 100%. The factor loadings of each factor are shown in Table 1.

**Table 1:** Exploratory factor analysis of dietary patterns

Food groups	n	%	Factor loadings	
			Factor 1: Healthy dietary pattern	Factor 2: High-fat-sugar-salt dietary pattern
Healthy food groups (never or < 1 time/week)				
Fish, Shrimp, Seafood	1218	6.58	**0.543**	0.130
Meat	553	2.99	**0.509**	0.136
Fruits	323	1.74	**0.453**	−0.153
Bean	1542	8.33	**0.440**	−0.083
Vegetables	246	1.33	**0.425**	−0.187
Egg	541	2.92	**0.421**	0.078
Dairy products	1027	5.55	**0.197**	0.053
Grains and Roots	39	0.21	**0.176**	−0.037
HFSS food groups (almost every day or every day)				
Beverages/ Coke / Soda	1479	7.99	−0.047	**0.592**
Hamburger/ Pizza/ Fried Chicken	90	0.49	0.036	**0.484**
Sweets/ Cookies/ Cakes	5024	27.14	0.023	**0.436**

*HFSS* High-fat-sugar-salt

The first factor was labeled as a healthy dietary pattern, which included the aforementioned first eight kinds of high-protein-and-high-fiber foods, and the score of this factor was constructed by averaging the frequencies of these eight food groups. The second factor was named as a high-fat-salt-sugar (HFSS) dietary pattern, which included the last three kinds of common unhealthy food groups which are high in fat, sugar, or salt [34]. Also, this factor score was constructed by averaging the frequencies of these three food groups (Table 1).

### Descriptive analysis

Table 2 shows the characteristics of the study sample. The children’s mothers had a mean educational level of 12.93 years (standard deviation (SD) = 2.62), and a mean household income of 3.91 units (SD = 1.29) which was about 600,000 to 1,000,000 NTD (1 USD ≒ 30 NTD) per year. The average score of healthy dietary pattern was 3.35 (SD = 0.4) indicating that the sample children consumed healthy foods about three to five times a week, while the average score of HFSS dietary pattern was 1.93 (SD = 0.65) showing that they consumed unhealthy foods about once or twice a week. The average level of children’s physical aggression at age 5 was 1.68 (SD = 0.74) suggesting a low level, while the level of aggression was a little higher at age 3 (mean = 2.04, SD = 0.87). The mean score of the children’s mental distress was 1.54 (SD = 0.61), indicating a low level of mental distress at age 5.

**Table 2:** Descriptive statistics of study variables (complete data, *n* = 18,513)

	Mean	SD	Minimum	Maximum
Maternal education (6 months)	12.93	2.62	0.00	17.00
Healthy dietary pattern (5 years old)	3.35	0.40	0.50	4.00
High-fat-sugar-salt dietary pattern (5 years old)	1.93	0.65	0.00	4.00
Physical aggression (5 years old)	1.68	0.74	1.00	5.00
Physical aggression (3 years old)	2.04	0.87	1.00	5.00
Mental distress (5 years old)	1.54	0.61	1.00	5.00
Household income (6 months)	3.91	1.29	1.00	8.00
	n	%		
Boys	9718	52.49		
Preschoolers living with married parents	16,785	90.67		

Missing numbers: 6 of marital status, 595 of maternal education, 186 of income, 1 of healthy dietary pattern, 2 of high-fat-sugar-salt dietary pattern, 535 of aggression at 3 years old, 4 of mental distress, 4 of aggression at 5 years old

Table 3 presents the bivariate correlations among continuous study variables. Maternal education is positively associated with children’s healthy dietary pattern, household income, and mental distress, and negatively associated with an HFSS dietary pattern, and physical aggression at ages 5 and 3. At the same time, the healthy dietary pattern was negatively associated with physical aggression at ages 5 and 3, and mental distress, and positively associated with household income. Also, the HFSS dietary pattern was positively associated with physical aggression at ages 5 and 3, was not associated with mental distress, and was negatively associated with household income. Physical aggression at ages 5 and 3 are highly positively correlated with each other, have a weak positive association with mental distress, and are negatively associated with household income.

**Table 3:** Bivariate correlations among maternal education, dietary patterns, physical aggression and related factors (*n* = 18,513)

	Maternal education	Healthy dietary pattern	High-fat-sugar-salt dietary pattern	Physical aggression	Physical aggression	Mental distress
Maternal education (6 months) (Range: 0–17)	1.000					
Healthy dietary pattern (5 years old) (Range: 0–4)	0.170 ***	1.000				
High-fat-sugar-salt dietary pattern (5 years old) (Range: 0–4)	− 0.216***	−0.007	1.000			
Physical aggression (5 years old) (Range: 1–5)	− 0.103***	− 0.090***	0.170***	1.000		
Physical aggression (3 years old) (Range: 1–5)	−0.126***	−0.078***	0.149***	0.381 ***	1.000	
Mental distress (5 years old) (Range: 1–5)	0.036***	−0.029***	0.003	0.104 ***	0.042 ***	1.000
Household income (6 months) (Range: 1–8)	0.527***	0.194***	−0.164***	− 0.098 ***	− 0.105 ***	0.002

***: *p* < 0.001

### Mediation analysis

Figure 1 and Table 4 demonstrate the relative total effect of maternal education on children’s physical aggression, considering dietary patterns and covariates. Children with mothers having a higher level of education showed less physical aggression (path C: B = -0.011, *p* = < .001). The mediation effects of children’s healthy dietary pattern and HFSS dietary pattern on the associations between maternal education and children’s physical aggression are also shown. Results indicate that maternal education was positively associated with children’s healthy dietary pattern (path a_1_: B = 0.014, *p* = 0.002) which was negatively associated with their physical aggression (path b_1_: B = − 0.096, *p* = 0.013). The indirect effect of maternal education on children’s physical aggression through the healthy dietary pattern was established (a_1_˟b_1_ = − 0.001, *p* < .001, Bias-corrected bootstrap 95% CI: − 0.002, − 0.001). On the other hand, maternal education was negatively associated with children’s HFSS dietary pattern (path a_2_: B = -0.042, p = 0.002), which was positively associated with children’s physical aggression (path b_2_: B = 0.123, *p* = 0.008). The indirect effect of maternal education on children’s physical aggression through the HFSS dietary pattern was also confirmed (a_2_˟b_2_ = − 0.005, p = < .001, Bias-corrected bootstrap 95% CI: − 0.006, − 0.004). Dietary patterns acted as partial mediators since they accounted for some, but not all, of the relationship between maternal education and children’s physical aggression. The direct effect of maternal education on children’s physical aggression was suppressed but still moderately significant (path c’: B = − 0.005, *p* = 0.036). Approximately 18% of the variance for preschoolers’ physical aggression can be explained by the mediation model (R-square = 0.178).

**Table 4:** Results for the mediation analysis (*n* = 18,513)

		M1: Healthy dietary pattern				M2: High-fat-sugar-salt dietary pattern				Y: Physical aggression		
Direct effects		Coeff.	SE	P		Coeff.	SE	P		Coeff.	SE	P
Constant		3.076	0.026	<.001		2.413	0.032	<.001		1.022	0.054	<.001
X: Maternal education (6 months)	a_1_	0.014	0.002	<.001	a_2_	−0.042	0.002	<.001	c’	− 0.005	0.002	0.036
M1: Healthy dietary pattern (5 years old)									b_1_	−0.096	0.013	<.001
M2: High-fat-sugar-salt dietary pattern (5 years old)									b_2_	0.123	0.008	<.001
Cov 1: Child sex (ref: girl) (6 months)		0.008	0.006	0.157		0.008	0.009	0.405		0.120	0.010	<.001
Cov 2: Married parents (ref: not-married) (6 months)		−0.002	0.010	0.839		−0.013	0.016	0.437		0.057	0.017	0.001
Cov 3: Physical aggression (3 years old)		−0.024	0.003	<.001		0.090	0.005	<.001		0.291	0.006	<.001
Cov 4: Mental distress (5 years old)		−0.020	0.005	<.001		0.004	0.008	0.574		0.109	0.008	<.001
Cov 5: Household income (6 months)		0.044	0.003	<.001		−0.032	0.004	<.001		−0.017	0.005	<.001
		R^2^ = 0.048				R^2^ = 0.065				R^2^ = 0.178		
		F(6, 18,506) = 154.34, p < .001				F(6, 18,506) = 213.21, p < .001				F(8, 18,504) =500.64, p < .001		
		Effect	Bootstrap SE	Bootstrap 95% CI								
Total effect of X on Y	c	−0.011	0.002	−0.016	−0.007							
Direct effect of X on Y	c’	−0.005	0.002	−0.009	0.000							
Indirect effect of X on Y		Effect	Bootstrap SE	Bootstrap 95% CI								
M1: Healthy dietary pattern (5 years old)	a_1_ × b_1_	−0.001	0.000	−0.002	−0.001							
M2: High-fat-sugar-salt dietary pattern (5 years old)	a_2_ × b_2_	−0.005	0.000	−0.006	−0.004							

a_1_ and a_2_ = effects of maternal education on healthy dietary pattern and high-sugar-fat-salt dietary pattern

b_1_ and b_2_ = effects of healthy and high-sugar-fat-salt dietary patterns on physical aggression

C = total effects of maternal education on physical aggression

C′ = direct effect of maternal education on physical aggression after adjustment for dietary patterns and covariates

a_i_ × b_i_ = mediation effects of dietary patterns on the relationship between maternal education and physical aggression

*CI* confidence interval

*X* independent variable

*Mi* mediators

*Cov* covariates

## Discussion

Our findings show significant associations between dietary patterns and physical aggression among preschoolers, and maternal education level is an antecedent of dietary patterns and physical aggression. These results indicate that high maternal education level increases the score of a healthy dietary pattern and decreases the score of an HFSS dietary pattern, which in turn decreases the level of preschoolers’ physical aggression. Dietary patterns may play an important role as mediators, and can be manipulated to intervene in the association between maternal education and physical aggression. The direct association between diet and aggression implies that encouraging preschoolers to have a healthy dietary pattern and avoid an unhealthy dietary pattern can protect them from physical aggression development.

We found a partial mediation effect of dietary pattern between maternal education and physical aggression among preschoolers, which reveals that dietary patterns partially intervene in the relationship between them. To the best of our knowledge, this is the first study that identifies a pathway showing that maternal education affects preschoolers’ dietary patterns, which in turn influences their physical aggression. It should be noted that a previous study discovered that low zinc levels and low maternal education were associated with externalizing behaviors, respectively; however, the study did not consider the mediation effect of diet [35]. Our study suggests that greater attention to the education of parents about a healthy dietary pattern is probably a practical way for physical aggression prevention in the preschool period.

Based on our EFA result, two dietary patterns were extracted which are similar to the results of Ambrosini et al.’s study which retrieved two dietary patterns, the healthy and the Western pattern, among Australian adolescents [32]. The food groups used for EFA were selected by the research team following the instrument development process and can represent common foods rather than all kinds of foods among preschoolers. A healthy dietary pattern signifies a balanced diet which includes foods from the six essential food groups. A healthy diet has profound influences for young children in the preschool period because healthy eating behaviors not only affect their health status but also affect their development of externalizing problem behaviors [35, 36]. The sufficient nutrients provided by these food groups, such as minerals and vitamins, have a positive influence on children’s moods [10, 37].

We found that high intakes of HFSS foods are positively associated with physical aggression. This result is consistent with other studies which emphasized problem behaviors, such as attention deficit hyperactivity disorder, depressive symptoms, and autism [13, 14, 34, 38, 39]. In addition, a previous study which investigated the one-by-one associations between various junk foods and violent behaviors also found a positive association between daily consumption of salty snacks and children’s physical fighting [34]. Intriguingly, compared to healthy foods, our result indicates that HFSS foods affect preschooler’s physical aggression to a greater extent. The mechanism between unhealthy dietary pattern and physical aggression might be the imbalanced intake of nutrients because a diet consisting of HFSS foods usually contains insufficient nutrients related to syntheses of neurotransmitters which may affect children’s mood [10]. On the other hand, the HFSS foods, usually highly processed, might contain high levels of trans fatty acids which might lead to a higher possibility of manifesting physical aggression [40].

The quality of preschoolers’ diet can be affected by family environment, which is usually constructed by mothers [17]. In this study, we found that maternal education directly links to preschoolers’ dietary patterns. The results echoed previous literature that a higher level of maternal education increases children’s healthy eating behavior [17, 20, 22]. Recent empirical evidence shows that mothers with a higher level of education had a higher probability of providing high-nutrient foods (e.g., cereals, dairy products, fruits, and vegetables) for their preschoolers instead of low-nutrient foods (e.g., sweetened beverages, fast foods, salty snacks, and sweets) [21, 36, 41]. Well-educated mothers will provide a better home food environment in many ways, including increasing the healthy food availability at home and setting food consumption roles, and this will increase children’s healthy eating behavior [20].

Indeed, maternal education level is not only related to their children’s eating behaviors but is also associated with their physical aggression [16, 42]. Mothers with a higher education level affect preschoolers’ physical aggression via using contemporary knowledge about parenting to educate and discipline their children, and this will subsequently prevent and protect them from developing problem behaviors [42]. For mothers with low levels of education, special attention should be paid, and the provision of some services, such as non-mother childcare services, is suggested for reducing exposure to risks in the family environment [42].

### Strengths and limitations

The main strength of this study is the large national representative sample, which can reflect the phenomena of Taiwanese preschoolers. Due to the sampling strategy used in this study, the results can be generalized to the population of preschoolers in Taiwan. The other strength is the long-term follow-up design that more clearly indicates the temporal sequence between maternal education and behavioral outcomes. Although this study controlled for baseline aggression and other related factors including household income, parental marital status, and preschoolers’ mental health to reduce the effect of possible confounding factors, there are still some other confounders that were not considered, for example, knowledge of healthy eating and parenting skills. In addition, because the dietary patterns and physical aggression were measured in the same year, the results cannot rule out the possibility that maternal education impacts on dietary patterns via physical aggression among preschoolers. Besides, the sample size for the two-week test-retest reliability for the measurement of aggression and mental distress is relatively small, although the coefficients of the reliabilities are quite acceptable (both > 0.7). Furthermore, since 97% of children went to daycare in the day time, the information about their problem behaviors and food consumption while in daycare might be unknown to their mothers. Last, the study cannot exclude the possibilities that the reporters might have underreported the frequencies of preschoolers’ physical aggression and unhealthy dietary behaviors. Despite these limitations, this study reveals the mediating roles of preschoolers’ dietary patterns between maternal education and physical aggression, and sheds light on the importance of nutritional education for mothers with preschoolers.

## Conclusions

Our findings support the proposition that dietary patterns as manipulable factors can mediate the association between maternal education and preschoolers’ physical aggression; however, only slightly partial mediation effects were found. The pathway through a healthy dietary pattern may result in lowering the level of physical aggression, while the pathway through an HFSS dietary pattern may increase the level of physical aggression. Thus, it is suggested that nutritional education for poorly-educated mothers might be an effective intervention strategy for decreasing or preventing their preschooler’s physical aggression. However, future interventional studies may be needed to examine the protective effect of a healthy balanced diet on physical aggression, especially among preschoolers raised by poorly-educated mothers.

## Data Availability

The datasets used and analysed during the current study are available from the corresponding author on reasonable request.

## Abbreviations

HFSS: High-fat-sugar-salt
TBCS: Taiwan Birth Cohort Study
EFA: Exploratory Factor Analysis
SMC: Squared multiple correlations
CI: Confidence interval
SD: Standard deviation
